# Incidental brain tumor findings in children: prevalence, natural history, management, controversies, challenges, and dilemmas

**DOI:** 10.1007/s00381-024-06598-z

**Published:** 2024-08-31

**Authors:** Jehuda Soleman, Shlomi Constantini, Jonathan Roth

**Affiliations:** 1 Department of Neurosurgery and Pediatric Neurosurgery, University Hospital and Children’s Hospital Basel, Spitalstrasse 21, Basel, 4031 Switzerland; 2 Faculty of Medicine, University of Basel, Basel, Switzerland; 3 Department of Pediatric Neurosurgery, Dana Children’s Hospital, Tel Aviv Medical Center, Tel Aviv-Yafo, Israel

**Keywords:** Incidentalomas, Pediatric brain tumors, Incidental brain tumors, Low grade gliomas, Malignant transformation, Pediatric neurosurgery, Pediatric neurooncology

## Abstract

Incidental brain tumor findings in children involve the unexpected discovery of brain lesions during imaging for unrelated reasons. These findings differ significantly from those in adults, requiring a focus on pediatric-specific approaches in neurosurgery, neuroimaging, and neuro-oncology. Understanding the prevalence, progression, and management of these incidentalomas is crucial for informed decision-making, balancing patient welfare with the risks and benefits of intervention. Incidental brain tumors are observed in about 0.04–5.7% of cases, with most suspected low-grade lesions in children showing a benign course, though up to 3% may undergo malignant transformation. Treatment decisions are influenced by factors such as patient age, tumor characteristics, and family anxiety, with conservative management through surveillance often preferred. However, upfront surgery may be considered in cases with low surgical risk. Initial follow-up typically involves a comprehensive MRI after three months, with subsequent scans spaced out if the lesion remains stable. Changes in imaging or symptoms during follow-up could indicate malignant transformation, prompting consideration of surgery or biopsy. Several challenges and controversies persist, including the role of upfront biopsy for molecular profiling, the use of advanced imaging techniques like PET-CT and magnetic resonance spectroscopy, and the implications of the child’s age at diagnosis. These issues highlight the need for further research to guide management and improve outcomes in pediatric patients with incidental brain tumor findings.

## Introduction

**Fig. 1:**
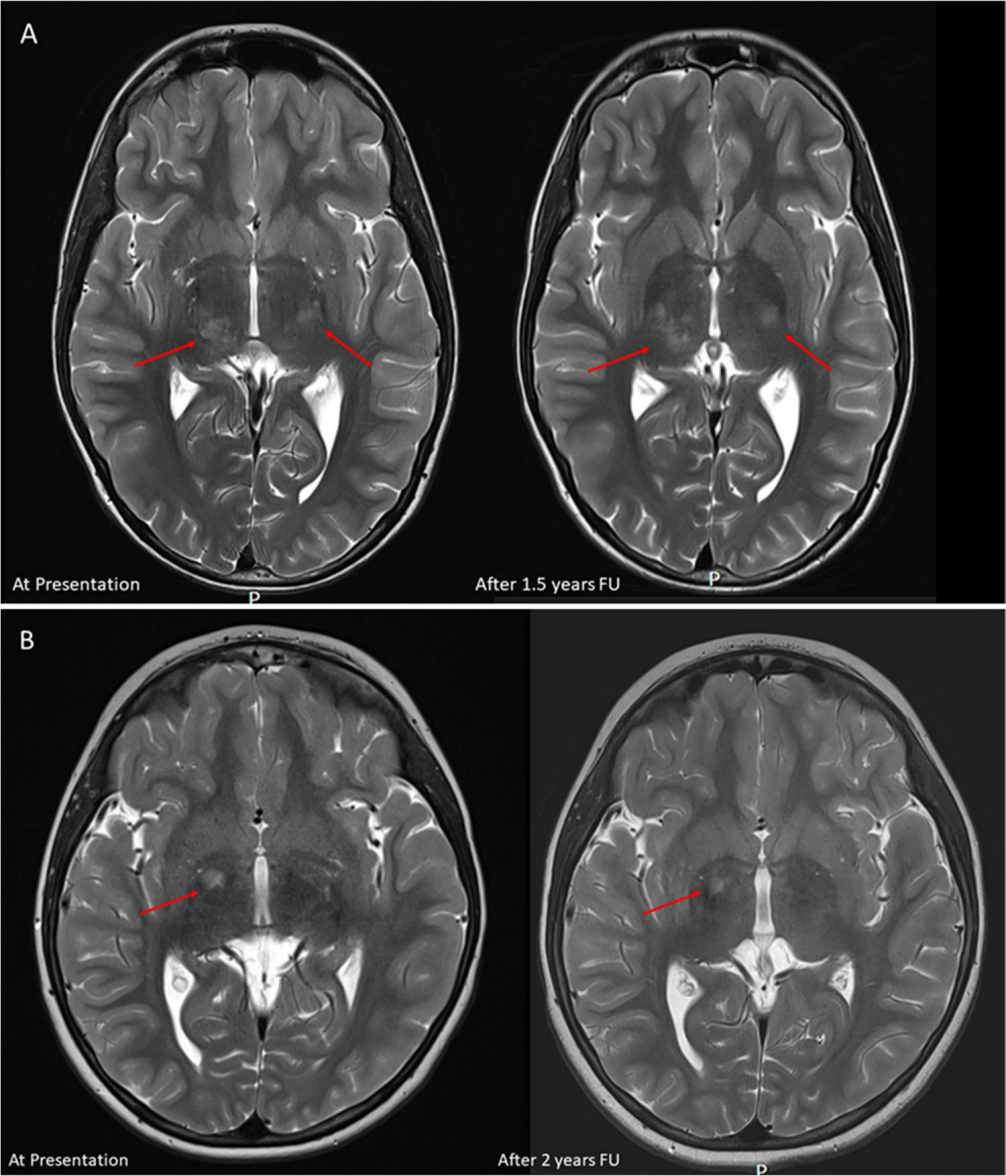
Examples of supratentorial incidental brain tumor findings managed conservatively **A** 12-year-old boy presenting with headaches. On MRI bi-thalamic incidental lesion. Conservative management with wait scan follow up was initiated, showing a stable lesion after 1.5 years. The boy remains neurologically intact **B** 7 year old girl presenting with scoliosis and torticollis. MRI showed an incidental finding in the right globus pallidus. Conservative management with wait scan follow up was initiated, showing a stable lesion after 2 years. The girl remains neurologically intact

**Fig. 2:**
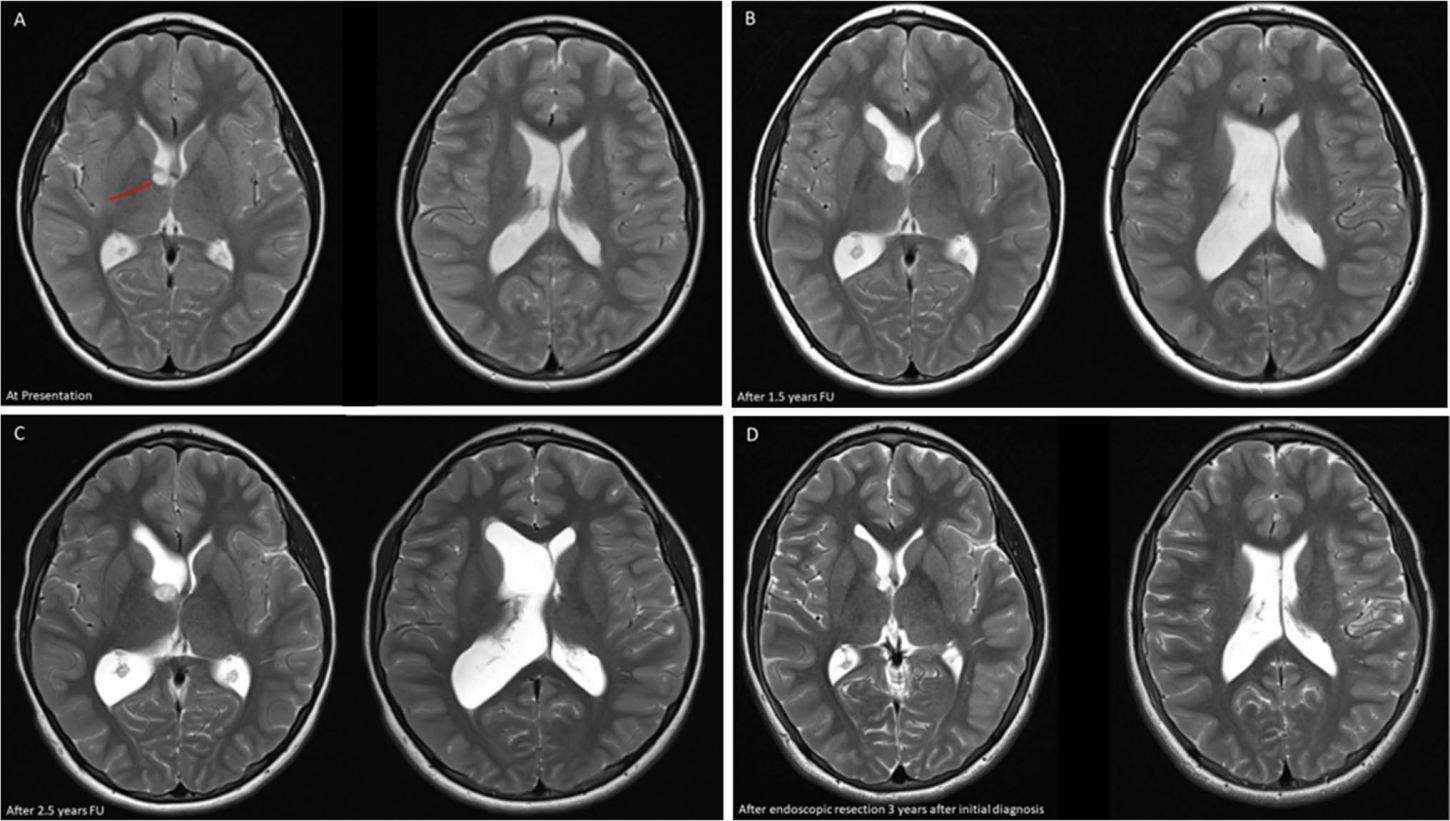
Example of a supratentorial incidental brain tumor finding eventually managed surgically **A** 11 year old boy presenting with growth retardation. On MRI paraventricular incidental finding within the basal ganglia. Initially a conservative management with wait scan follow up was initiated **B** and **C** Due to minimal growth of the lesion and clear progression of the ventricular size, an endoscopic transventricular removal of the lesion was undertaken, showing a low grade glial/glioneuronal tumor **D** 3 years postresection no recurrence or residual tumor is seen

**Fig. 3:**
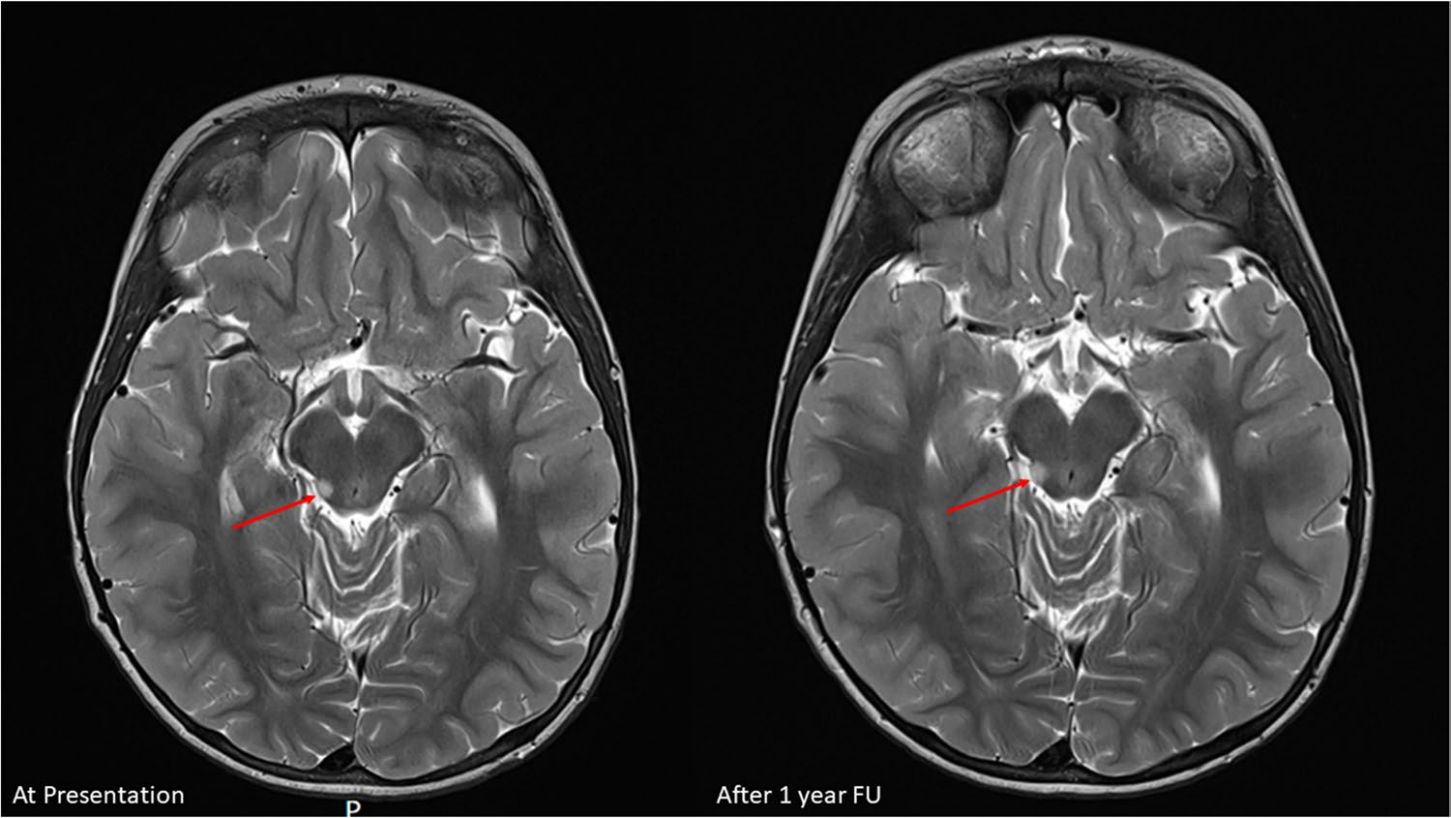
Example of a brain stem incidental brain tumor finding 10-year-old boy presenting with growth retardation. On MRI incidental finding in the right posterior mesencephalon. Conservative management with wait scan follow up was initiated, showing a stable lesion after 1 year. The boy remains neurologically intact

Incidental brain tumor findings in children refer to the unintentional discovery of abnormal growths or lesions in the brain during medical imaging procedures performed for reasons unrelated to suspected neurological issues in the pediatric population (Figs. 1, 2 and 3). The characteristics and implications of Incidental brain tumor findings in children differ from those in adults, requiring a specific focus on the unique aspects of pediatric neurosurgery, pediatric neuroimaging, and pediatric neuro-oncology [1, 2]. These incidental findings can encompass a spectrum of lesions, including both non-neoplastic and neoplastic growths [3]. Non-neoplastic lesions may include developmental anomalies, cysts, or vascular malformations, while neoplastic lesions, referred to as incidentalomas, may range from benign tumors to malignancies. Incidentalomas pose a unique set of challenges, as the clinical significance of these tumors varies widely [1, 4–6]. Some incidentalomas may be benign, asymptomatic, and require no immediate intervention, while others may be malignant or have the potential to cause neurological symptoms, necessitating careful management and consideration of potential treatment options (Figs. 1, 2 and 3). Understanding the prevalence, natural history, and appropriate management of incidentalomas is crucial to make informed decisions that prioritize patient well-being and balance the potential risks and benefits of an intervention [1]. In this manuscript, we provide a comprehensive overview of the documented prevalence and natural history of incidentalomas in children. Additionally, we delve into the various management strategies, highlighting existing controversies, challenges, and dilemmas surrounding the treatment of these patients.

## Prevalence of incidentalomas in children

Due to the constantly growing availability of neuroimaging, an increase in incidental brain findings is observed. To date, incidental brain findings are noted in approximately 20–25% of preformed brain imaging in children [1, 3]. While most incidental findings in children comprise cysts (e.g., pineal/arachnoid cyst, 10%), non-specific white matter lesions (1.6%), tonsillar ectopia (0.4%), ventriculomegaly/hydrocephalus (0.3%), Chiari malformation (0.2%), and cavernous malformations (0.05%), incidental brain tumors are observed in only around 0.04–5.7% of cases [1, 3, 7, 8]. It is noteworthy that incidentalomas in children are overall less frequent and significantly differ from those in their adult counterparts [2]. In adults, meningiomas, pituitary lesions, non-glioma neoplastic lesions, and vascular lesions are frequently observed; however, these pathologies are very rare in children [2]. In the future, it is anticipated that the prevalence of incidental brain findings, including incidentalomas, will continue to increase, due to the growing availability of modern imaging techniques.

## Natural history of incidentalomas in children

In incidentalomas, the natural history is clearly strongly influenced by the underlying pathology and molecular profile of each specific lesion [1, 6, 9–11]. Additionally, factors such as cancer predisposition syndromes (e.g., neurofibromatosis type I/II, tuberous sclerosis, Li-Fraumeni, Turcot, von Hippel-Lindau, and Gorlin syndrome), previous radiation treatments, and the location of the lesion may play a role in the natural history of the lesion [1, 12, 13]. Most studies analyzing pediatric patients indicate that for suspected benign incidentalomas, the natural history is often favorable, showing rare progression or malignant transformation of the lesion [1, 4, 6, 9, 14]. Some reports even demonstrate regression of these lesions over time [4]. This is in strong contrast to the adult population, where the progression of low-grade benign lesions is frequently observed, and malignant transformation occurs in up to half of cases [1, 4]. Unfortunately, due to the low prevalence, only a few studies exist describing the natural history of incidentalomas in children, and large prospective trials are lacking. Based on the available literature, it seems, however, that in children, incidentally detected lesions suspected to be of low-grade nature usually exhibit a benign course, remaining stable or regressing and, therefore, often do not necessitate surgical or oncological treatment [1, 4].

## Diagnosis and Radiological features of incidentalomas in children

To date, comprehensive studies examining and analyzing radiological features on magnetic resonance imaging (MRI) at a large scale, specifically aimed at distinguishing between low-grade and high-grade lesions, are lacking. From the existing literature, we understand that benign lesions typically manifest as hypo- or isointense on T1 weighted imaging (WI) and hyperintense on T2WI. Furthermore, low-grade lesions typically exhibit minimal or heterogeneous contrast agent uptake, while high-grade lesions often display ring-enhanced or more uniformly enhanced contrast patterns. Additionally, features such as diffusion restriction on diffusion-weighted imaging, as well as surrounding edema or mass effect, are seldom observed in low-grade lesions [1, 11]. Nevertheless, the absence of definitive radiologic criteria to differentiate between low-grade and high-grade lesions continues to fuel considerable debate regarding the accuracy of radiologic diagnostics and surveillance. Magnetic resonance spectroscopy (MRS) has proven advantageous in distinguishing non-neoplastic lesions from both low-grade and high-grade tumors [15–19]. In the differential diagnosis of the three most frequent pediatric brain tumors (pilocytic astrocytoma, medulloblastoma, and ependymoma) the combination of different types of MRS showed a diagnostic accuracy of up to 98% [20]. Despite suggestions that MRS surpasses conventional MRI in the differentiation of brain tumors, the absence of prospective studies on pediatric incidentalomas prevents us from conclusively determining the applicability of MRS in the pediatric population. The indication of low or negligible tracer uptake on positron emission tomography (PET) has been proposed as a valuable discriminator between low-grade brain tumors and their malignant counterparts [21, 22]. Radiomics, machine learning, and artificial intelligence are under scrutiny to ascertain whether these tools can enhance the diagnosis of incidental lesions and distinguish between various tumor types. Promising results have been observed in small-scale studies; however, these findings must undergo evaluation in large prospective cohorts before they can be integrated into routine clinical practice [23–27]. Similarly, liquid biopsies are undergoing assessment for various pediatric tumors, including brain tumors. Nevertheless, the diagnostic yield and precision continue to be unclear, accompanied by the ongoing challenges of high costs and effort, especially given that the biopsy often relies on cerebrospinal fluid samples [28–30].

## Management options of incidentalomas in children

The management options for incidentalomas in the pediatric population encompass various strategies, including clinical and radiological surveillance (“wait and watch”), open or stereotactic biopsy, upfront treatment with radiotherapy and/or chemotherapy, or surgical resection [1, 5, 31]. However, definitive guidelines with clear criteria for selecting the appropriate treatment pathway are currently lacking. Decision-making remains a case-by-case process, guided by interdisciplinary discussions within a pediatric tumor board. Here, consensus is reached on the recommended treatment, followed by shared decision-making with the patient and the parents.

Numerous factors influence treatment decisions, including parental and child anxiety, patient age, radiological characteristics, tumor size and location, tumor predisposing syndromes, presenting symptoms, as well as the preferences and experiences of the medical team.

Traditionally, conservative management with clinical and radiological surveillance has been favored, particularly as malignant transformation in children was considered rare, with most studies indicating a benign course for these lesions [1, 4–6, 32–35]. However, recent reports have highlighted the possibility of malignant transformation in approximately 3% of cases, with emerging risk factors identified through molecular biology studies [6, 36–40]. Consequently, the debate regarding early or even upfront resection or biopsy has resurfaced. Nonetheless, given the rarity of malignant transformation and most cases remaining stable or regressing (65–75%), conservative treatment is generally recommended [1, 4]. Clear guidelines for the follow-up regimen in these patients are currently lacking. Typically, follow-up intervals are determined based on factors such as the natural progression of the lesion, its location, and radiological characteristics. Generally, most authors recommend an initial scan after three months, comprising a comprehensive central nervous system MRI (brain and whole spine). Subsequently, the frequency of scans can be gradually extended, such as spacing them six months apart after one year of follow-up, and then to annual intervals if the lesion remains stable or shows regression [1, 5]. For future scans, such as those scheduled after five years, the timing can be adjusted based on the duration of stability, radiological findings, location of the lesion, and collaborative decision-making within the medical team, as well as with the patient and their parents. However, meticulous evaluation of follow-up images by specialized teams, including volumetric measurements of lesion size, and careful monitoring of any changes in radiological parameters over time, is crucial for guiding treatment decisions. Any changes observed over time should prompt interdisciplinary discussions within the treating team, leading to shared decision-making with the patient and their parents [5, 10, 11, 41].

Indications for upfront resection or biopsy are typically reserved for symptomatic lesions or those showing radiological features suggestive of malignancy (e.g., diffusion restriction, ring enhancement). Progression observed during follow-up may warrant consideration for resection, although a significant proportion of cases (62%) demonstrate no symptoms or eventual cessation of growth, indicating safe continued observation [1, 4]. Radiological changes or new symptoms suggestive of tumor progression should prompt consideration for surgical intervention or biopsy, as these could indicate malignant transformation. The decision for complete resection or biopsy depends largely on the lesion’s location (deep vs. superficial), its proximity to eloquent brain regions, and the associated surgical risks.

Upfront chemotherapy or radiotherapy is generally reserved for incidentally detected diffuse intrinsic pontine gliomas (DIPG) or bithalamic gliomas, where diagnosis can be established based on MRI findings [42–44]. However, the role of biopsy in these cases remains subject to ongoing debate, primarily aimed at gathering molecular genetic information for potential future treatments which should generally be done only within the framework of study protocols [36, 43–45]. Typically, in cases where progression is evident or malignancy is suspected, surgical resection or biopsy is recommended prior to initiating chemotherapy or radiotherapy.

## Controversies, challenges and dilemmas

### Upfront resection or “wait and watch”?

The diagnosis of suspected incidentalomas in children poses a challenge due to the variability of lesions, which may include hamartomas, dysplasias, or inflammatory changes. Additionally, there are no definitive radiological criteria to distinguish between benign and malignant lesions. Unlike in adults, where early resection of suspected low-grade gliomas has been associated with improved long-term outcomes and reduced rates of malignant transformation, evidence supporting early resection in children is lacking [46–52]. While some studies suggest that the extent of resection is a significant factor in childhood low-grade gliomas, the relevance of early surgery with the goal of achieving gross total resection (GTR) for incidentalomas remains uncertain [5, 41]. Although resection of incidentalomas in adults has shown low morbidity rates and a low risk of secondary epilepsy, similar studies in the pediatric population are limited. Some authors advocate for upfront resection of incidentalomas in children to minimize psychological burden, given their longer life expectancy and the favorable long-term outcome of surgery [7]. However, the decision to proceed with upfront resection presents a dilemma in most cases.

Based on existing literature and considering the potential low risk of malignant transformation in children, the decision to opt for upfront resection of an incidentaloma is ultimately based on individual risk assessments. For superficial, non-eloquent seated lesions where surgical risk is likely lower than the risk of developing neurological symptoms, progression, or malignant transformation, upfront resection may be a viable option to achieve long-term positive outcomes [1, 4, 5]. However, in deep or eloquent seated lesions, where surgical risk outweighs the risk of developing neurological complications, progression, or malignant transformation, surgical resection should not be considered as the primary treatment approach [1, 4, 5].

### Does anatomical localization matter in the decision making?

Much of the literature examining the natural history, prevalence, and management of incidentalomas combines lesions from various regions of the brain. Studies have revealed that 40% of incidentalomas are situated within the posterior fossa, 58% in supratentorial regions, and 2% in the intraventricular space [4]. However, due to the diversity in anatomical localization and the limited sample size of anatomical sub-groups in existing literature, it is challenging to conduct a meaningful analysis regarding whether anatomical location influences progression rates, rates of malignant transformation, or clinical outcomes. Nevertheless, it is plausible that incidentalomas in different anatomical locations may harbor distinct pathologies that impact the natural history of the lesion. Therefore, there is a need for studies focusing on specific regions to gain a better understanding of the natural course of these lesions and to tailor treatment accordingly.

Our research group specifically analyzed two anatomical locations: posterior fossa and thalamic incidentalomas [10, 11]. Among the 70 posterior fossa incidentalomas, approximately 56% eventually underwent surgical resection (6 biopsy, 6 partial resection, and 27 gross total resection) over a mean follow-up period of 44 months. Immediate surgery was performed in 27 patients (39%), while 12 patients underwent resection after a period of observation. Malignant lesions were encountered in 10% of the surgically treated cases, and the presence of lesion enhancement and diffusion restriction at initial diagnosis or during follow-up was significantly associated with surgical intervention. Malignant transformation did not occur during the follow-up period [10]. Among the 58 patients with thalamic incidentalomas, 21 (36%) underwent surgery over a mean follow-up period of 60 months. Of these, 11 patients (19%) underwent immediate surgery, while an additional 10 patients (21% of the 47 followed patients) underwent delayed surgical treatment. Malignant lesions were observed in 14% (3 out of 21) of the operated cases, with suspected malignant transformation in one patient [11]. These preliminary results suggest slight differences in the natural history and rates of surgical treatment depending on the anatomical location. It is noteworthy that both locations demonstrated a malignancy rate of approximately 10%, with suspected cases of malignant transformation being rare. Further studies based on large multicenter cohorts, grouping and analyzing incidentalomas based on their anatomical location, are therefore warranted.

### Should we seek routine histological and molecular profiling of incidentalomas?

Whether routine histological and molecular profiling of incidentalomas in children should be pursued is a topic of ongoing debate and requires careful consideration. On one hand, obtaining histological and molecular profiles can provide valuable insights into the underlying pathology and molecular biology of these lesions, potentially aiding in treatment planning and prognosis assessment [53–55]. Additionally, molecular profiling may identify specific genetic mutations or biomarkers that could inform targeted therapies or clinical trial eligibility [36]. However, there are also potential drawbacks to routine histological and molecular profiling. Surgical treatment carries inherent risks, including complications such as bleeding or infection, which may outweigh the potential benefits, particularly if the lesion is asymptomatic or deemed low-risk based on imaging characteristics [1]. Furthermore, histological and molecular profiling may not always yield definitive diagnostic or prognostic information, and there is a risk of overdiagnosis or misinterpretation of findings. Molecular profiling holds the potential to predict the risk of malignant transformation in incidentalomas [37]. Studies have shown that certain genetic alterations, such as BRAF V600E mutation, CDKN2A deletion, H3K27M mutation, and TP53 alteration in low-grade gliomas (LGG), are associated with a higher risk of malignant transformation [36, 53, 56]. The emergence of these findings, coupled with reports of pediatric LGG undergoing malignant transformation, has reignited the debate surrounding the routine use of biopsy or surgical resection for molecular profiling.

At present, however, there is insufficient evidence to support the notion that molecular profiling in these lesions leads to significantly improved treatment outcomes or survival rates. Consequently, routine tissue sampling for molecular profiling cannot be recommended and should only be conducted within the framework of studies with predefined protocols. Nonetheless, if a BRAF V600E, CDKN2A, MMRD, or TP53 alteration is detected in an incidentaloma, adjustments to the follow-up and treatment approach should be made accordingly. A multidisciplinary approach involving pediatric neurosurgeons, pediatric oncologists, pediatric radiologists, and neuropathologists is essential to weigh these considerations and make informed decisions tailored to each patient’s specific circumstances.

### Tumor predisposition syndromes- a reason for more aggressive treatment?

Predisposing syndromes play a critical role in decision-making regarding incidentalomas in children, serving to both reassure the benign nature of a lesion and predispose a child to a malignant tumor, thereby necessitating earlier treatment [5, 6, 12, 13]. For instance, patients with Li-Fraumeni syndrome are predisposed to choroid plexus carcinoma and require early surgical intervention [57]. Likewise, Turcot syndrome predisposes individuals to malignant tumors such as medulloblastomas and GBM, warranting early surgical treatment [58]. On the other hand, an intra-axial enhancing mass detected in a child with neurofibromatosis type I (NF1) typically indicates a low-grade lesion until proven otherwise. Diagnosis can often be established based on MRI criteria, prompting the need for appropriate monitoring [42]. Even in cases where lesions of NF1 patients progress, biopsy is frequently unnecessary, and oncological or radio-oncological treatment can be initiated without prior histological assessment [59–62]. Similarly, in patients with tuberous sclerosis presenting with a ventricular enhancing lesion, a subependymal giant cell astrocytoma is a likely diagnosis. Management primarily involves surveillance or treatment with mTOR inhibitors, while surgical resection may eventually be required in certain cases [63]. In the management of incidentalomas in adult patients, predisposition syndromes seldom factor into decision-making, highlighting the distinction between pediatric and adult incidentalomas. This underscores the importance of specialized pediatric care teams when addressing pediatric incidentalomas.

### Does age at diagnosis matter?

According to existing literature, the average age at diagnosis of incidentalomas is approximately 10 years, ranging from one month to 18 years. Studies focusing on specific age groups are scarce due to the challenge of conducting meaningful analyses with limited sample sizes in age subgroups. Nonetheless, age significantly influences treatment decisions at various levels. Firstly, there is a recognized difference in histological diagnosis between neonates or infants and older children or teenagers with brain tumors [64]. Secondly, both the families of younger patients at the time of diagnosis and the patients themselves experience a heightened dilemma and burden of disease, attributed to the longer life expectancy of these patients. Thirdly, it remains uncertain whether malignant transformation, primarily documented in case reports across different age groups, occurs at similar rates throughout all age groups [1]. Lastly, the transition of patients into adulthood raises questions about whether their incidentalomas effectively become adult incidentalomas, warranting primary resection, or remain pediatric incidentalomas, where radiological and clinical stability dictates follow-up care. These aspects remain ambiguous to date and should be the focus of multicenter, large prospective trials.

### Medicolegal and ethical challenges

The management of pediatric incidentalomas presents complex medicolegal and ethical challenges. These include issues surrounding informed consent, shared decision-making, and ensuring the child’s best interests while respecting parental authority. There are concerns regarding the potential for overdiagnosis and overtreatment, as well as the psychological impact on the child and their family once an incidentaloma is diagnosed. Moreover, finding the equilibrium between aggressive intervention and conservative approaches presents clinicians with dilemmas, especially when weighing the potential long-term impact on the child’s quality of life and the burden it places on the patients and/or their parents. Ensuring equitable access to care and considering the socioeconomic and religious implications of treatment decisions are also crucial aspects of ethical considerations in pediatric incidentaloma management. Ultimately, a multidisciplinary approach, clear communication, and adherence to ethical guidelines are essential for addressing these challenges and providing optimal care for these pediatric patients.

## Conclusion

Pediatric incidentalomas are rare discoveries, although their prevalence is anticipated to increase in the coming years due to the availability of modern imaging techniques. The primary approach to suspected benign incidental brain tumor findings in children is typically conservative, as most lesions demonstrate no progression or even regress during follow-up, with malignant transformation being exceedingly uncommon. However, recent reports of malignant transformation in children, previously believed to be almost non-existent, have led to a growing number of authors, including our group, recommending early resection of incidentalomas if the surgical risk is low (such as in cases of superficial, non-eloquent lesions). The identification of specific molecular alterations within low-grade lesions associated with malignant transformation further supports these recommendations. Nonetheless, routine molecular profiling is not advised and should only be undertaken within the context of large-scale studies.

Any alterations in radiological features observed during follow-up warrant serious consideration and should prompt discussions regarding potential changes in treatment strategies. Numerous dilemmas, controversies, and challenges persist in the management, medicolegal, and ethical aspects of pediatric incidentalomas, many of which are likely to persist indefinitely. Therefore, shared decision-making involving the patient and/or their family is crucial, as is thorough discussion within a specialized pediatric care team, including pediatric oncologists, pediatric neurosurgeons, pediatric neurologists, pediatric neuroradiologists, and pediatric neuro-oncologists.

Future large multicenter studies, likely conducted under the auspices of international pediatric neurosurgical societies, are essential to advance understanding of pediatric incidental brain tumor findings.

## Data Availability

No datasets were generated or analysed during the current study.
